# B Cells in Rheumatoid Arthritis：Pathogenic Mechanisms and Treatment Prospects

**DOI:** 10.3389/fimmu.2021.750753

**Published:** 2021-09-28

**Authors:** Fengping Wu, Jinfang Gao, Jie Kang, Xuexue Wang, Qing Niu, Jiaxi Liu, Liyun Zhang

**Affiliations:** ^1^ School of Basic Medical Sciences, Shanxi Medical University, Taiyuan, China; ^2^ Department of Rheumatology, Shanxi Bethune Hospital, Shanxi Academy of Medical Sciences, Tongji Shanxi Hospital, Third Hospital of Shanxi Medical University, Taiyuan, China; ^3^ Third Hospital of Shanxi Medical University, Shanxi Bethune Hospital, Shanxi Academy of Medical Sciences, Tongji Shanxi Hospital, Taiyuan, China

**Keywords:** B cells, rheumatoid arthritis, ACPA, RF, autoreactive, therapeutic approaches

## Abstract

Rheumatoid arthritis (RA) is a common, chronic, systemic autoimmune disease, and its clinical features are the proliferation of joint synovial tissue, the formation of pannus and the destruction of cartilage. The global incidence of RA is about 1%, and it is more common in women. The basic feature of RA is the body’s immune system disorders, in which autoreactive CD4^+^T cells, pathogenic B cells, M1 macrophages, inflammatory cytokines, chemokines and autoantibodies abnormally increase in the body of RA patients B cell depletion therapy has well proved the important role of B cells in the pathogenesis of RA, and the treatment of RA with B cells as a target has also been paid more and more attention. Although the inflammatory indicators in RA patients receiving B-cell depletion therapy have been significantly improved, the risk of infection and cancer has also increased, which suggests that we need to deplete pathogenic B cells instead of all B cells. However, at present we cannot distinguish between pathogenic B cells and protective B cells in RA patients. In this review, we explore fresh perspectives upon the roles of B cells in the occurrence, development and treatment of RA.

## B Cells in the Synovium of RA Patients

**Figure 1:**
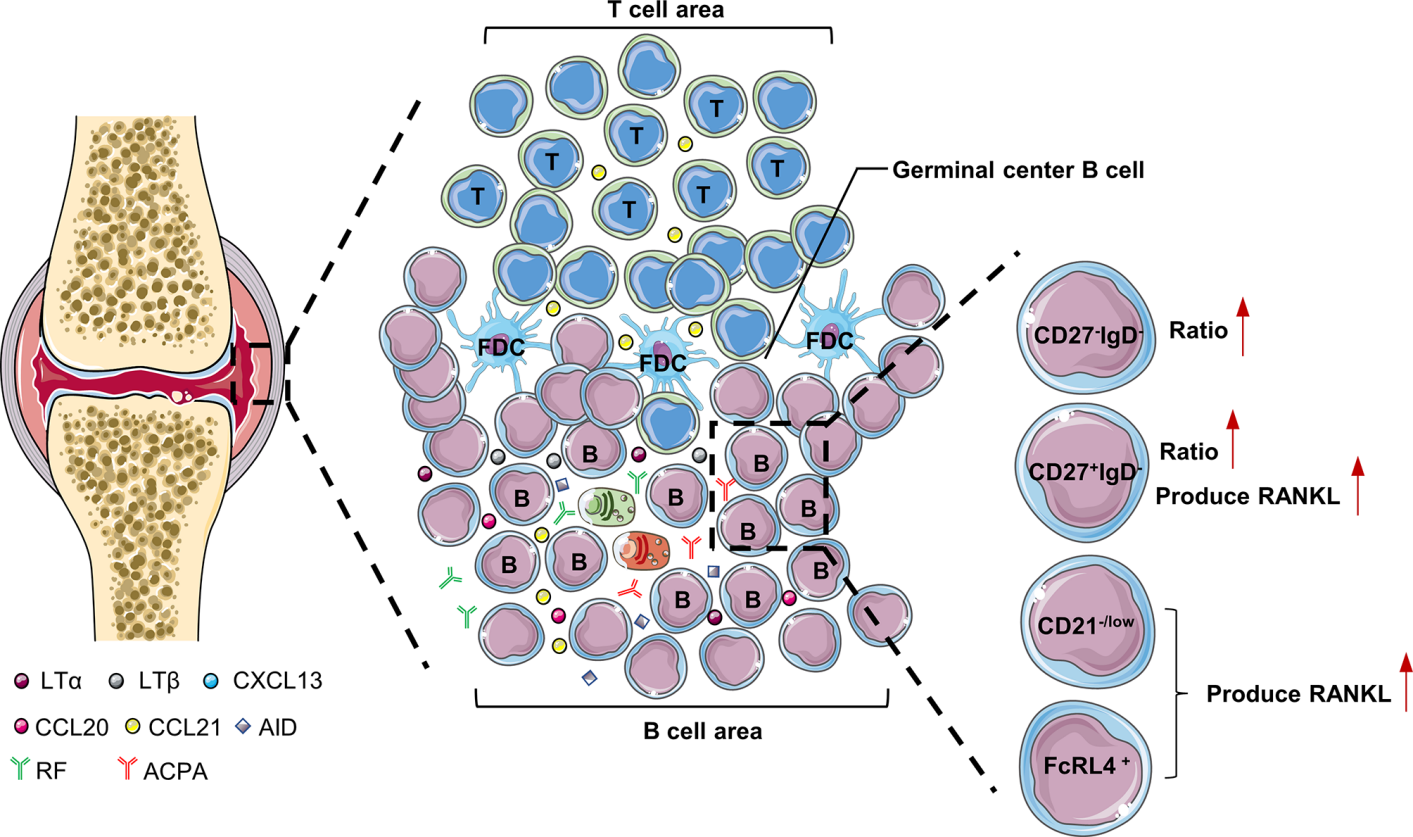
B cells in the synovium of RA. LTα and LTβ secreted by B cells are very important for maintaining the aggregated T cell and B cell infiltrate in the synovial tissue of RA patients. FcRL4^+^B cells, CD27^+^IgD^-^B cells and CD21^-/low^ B cells are prone to produce RANKL, which are pathogenic B cells in the synovium of RA patients. LTα, lymphotoxinα; LTβ, lymphotoxinβ; AID, Activation-induced cytidine deaminase; CXCL, C-X-C motif chemokine ligand; FcRL4, Fc receptor-like 4.

The synovial tissue of RA patients can be regarded as tertiary lymphoid tissues (TLTs) or ectopic lymphoid structures. Its structure is similar to secondary lymphoid tissue, with T cell and B cell differentiation sites. TLTs are correlated with autoantibody titers, inflammatory cytokine levels, and disease severity in RA patients, indicating that TLTs are related to the continuous inflammation in RA (1). In addition, accumulation of B cells in TLTs is related to the increase of radiographic scores and T cell activation in RA patients (2). There are abundant chemokines and inflammatory factors (LTα, LTβ, CXCL13, CCL20, CCL21, and CXCL12) in TLTs. These cytokines promote the infiltration of inflammatory cells into joints and the production of TLTs, which aggravates the formation of pannus and synovial hyperplasia (3). LTα and LTβ secreted by B cells are very important for maintaining the aggregated T cell and B cell infiltrate in TLTs (4). There are also plasmablasts that produce autoantibodies in TLTs, and even long-lived plasma cells (usually only in the bone marrow and are the main source of ACPA) (5). Interestingly, RA patients treated with etanercept (combined with TNF and LTα) had significantly fewer new TLTs in the synovium, which further illustrates the importance of B cells for TLTs production (6). TLTs have part of the functions of secondary lymphoid tissues, support B cell antigen presentation and interaction with T cells, and are conducive to the further differentiation and maturation of B cells. Studies have shown that B cells accumulated in the synovial membrane of RA have undergone somatic hypermutation (7). Activation-induced cytidine deaminase (AID) plays a major role in B cell somatic hypermutation and class switching recombination in TLTs (7). In addition to plasma cells, TLTs also have a large number of anti-citrullinated protein antibodies (ACPA) and rheumatoid factor (RF), so TLTs contribute to the production of autoantibodies in the synovium (8, 9) (**Figure 1**).

The results of single-cell sequencing showed that the ratio of double negative (CD27^-^IgD^-^) and class-switched memory (CD27^+^IgD^-^) B cells in the synovium of RA patients was significantly higher than that of peripheral blood, which shows these two types of B cell subgroups may play a key role in the pathogenesis of RA (10). Compared with other B cell subgroups, class-switched memory B cells (CD27^+^IgD^-^) are particularly prone to express RANKL after activation (11). Double-negative B cells often highly express miR-155, which is essential for B cells to produce autoantibodies (12, 13). In addition, there is a type of CD21^-/low^ B cells in the synovial fluid of RA patients with serum ACPA positive. CXCR3 is expressed on the surface of these cells, and RANKL can be secreted under the stimulation of IL-6 to induce osteoclast differentiation and ultimately cause bone destruction in RA patients (14). Obviously, CD21^-/low^ B cells belongs to pathogenic B cells and can be used as a potential target for the treatment of RA in the future.

## B Cell Checkpoints in RA

**Figure 2:**
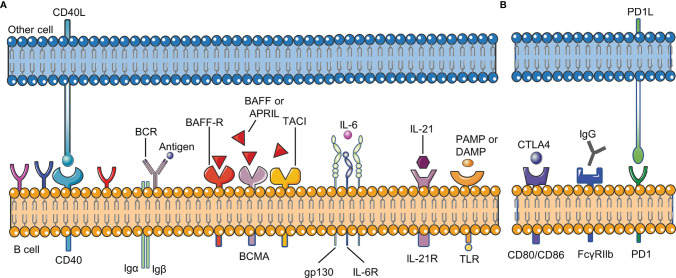
B cell checkpoints in RA. B cell checkpoints provide signals for the survival, development, differentiation, inhibition and other physiological processes of all B cells. **(A)** stimulatory checkpoint. **(B)** inhibitory checkpoint. BCR,B cell receptor; CD40L, CD40 ligand; BAFF, B cell activating factor; BAFFR, B cell activating factor receptor; DAMP, damage- associated molecular pattern; PAMP, pathogen- associated molecular pattern; FcγRIIβ, Fc gamma receptor IIβ; IL-21R, IL-21 receptor; IL-6R, IL-6 receptor; PD1, programmed cell death 1; CTLA4, cytotoxic T lymphocyte-associated antigen-4.

B cell checkpoints refer to a series of sites that affect the proliferation, differentiation, apoptosis and other physiological processes of all B cells during their development. These sites include receptors on the surface of B cells and their ligands (**Figure 2**).

### BCR, TLR, and CD40

Two signals are usually needed to activate B cells: BCR signal and costimulatory signal. The combination of antigen and BCR provides the first signal for B cell activation, and the costimulatory signal is important for B cells to overcome inhibitory checkpoints. TLR and CD40 on B cells are mainly responsible for providing costimulatory signals. In RA, BCR signaling has been proved to be involved in the development of autoreactive B cells (15). CD40L (CD40 ligand) on the activated T cell membrane will promote the formation of memory B cells and long-lived plasma cells. Without the co-stimulation of CD40 or other receptors, only activating the BCR signal will lead to B cell apoptosis. CD40L is significantly up-regulated in T cells in RA, and the level of soluble CD40L is correlated with autoantibody titers and disease activity in RA patients (16, 17). Clinical trials have shown that VIB4920 (a CD40L inhibitor) can inhibit the activation and differentiation of B cells and reduce the disease activity of RA patients (18). In addition, B cells can be activated in a T cell-independent manner by dual stimulation of BCR and TLR. TLR recognizes pathogen-associated molecular patterns (PAMP) and damage-associated molecular patterns (DAMP). TLR-7 and TLR-9 are necessary for the production of anti-RNA and dsDNA autoantibodies, respectively (19, 20). Studies have confirmed that the expression of TLR10 on B cell subsets in RA patients is related to disease activity, but the effect of TLR-10 on B cells needs further research (21).

### BAFF and APRIL

B-cell activating factor (BAFF) and A proliferation-inducing ligand (APRIL) are two members of the TNF superfamily. They have similar structures and are necessary for the growth and development of B cells (22, 23). BAFF and APRIL correspond to three types of receptors: B cell maturation antigen (BCMA), transmembrane activator and CAML interactor (TACI), BAFF-receptor (BAFF-R). BAFF-R is expressed on almost all B cells, and its importance for the survival of B cells is far greater than the other two receptors. Excessive BAFF in peripheral blood promotes the survival of autoreactive B cells and the production of autoantibodies (24). Studies have shown that compared with healthy individuals, the levels of BAFF and APRIL in the peripheral blood of RA patients are significantly higher; the levels of BAFF and APRIL in the synovial fluid of RA patients are also higher than those in the serum (25). BAFF and APRIL are constitutively expressed by various types of cells (including neutrophils, follicular dendritic cells, macrophages, and fibroblast-like synoviocytes) in RA patients, and their expression will be significantly increased in the inflammatory environment (25). The serum BAFF level of RA patients is positively correlated with the RF titer of seropositive RA patients, indicating that BAFF plays a key role in the occurrence or continuation of the disease (26). In the presence of BAFF, TLR ligands will promote B cell activation, class switching, somatic hypermutation and differentiation into plasma cells, leading to the production of harmful autoantibodies (27, 28). As a homologous of BAFF, APRIL can cause the accumulation of plasma cells in the joints and further increase the production of inflammatory cytokines such as TNF-α, IL-1 and IL-6 (29). Zhang LL et al. found that BAFF can promote B cell activation and differentiation through the NF-κB pathway, leading to the production of autoantibodies and inflammatory cytokines, and ultimately causing bone erosion and destruction in RA patients (26). In addition, inhibiting the expression of BAFF receptors will significantly reduce the proportion and number of B cells and the level of anti-collagen IgG in collagen-induced arthritis (CIA) mice, ultimately leading to a reduction in joint inflammation (30).

### IL-6

IL-6 was initially identified as a B cell growth factor and plasma cell differentiation factor, and was mainly produced by B cells and macrophages in the synovial fluid of RA patients (31, 32). Compared with healthy individuals, the concentration of IL-6 in serum and synovial tissue of RA patients is increased. In RA, increased serum concentration of IL-6 is associated with joint damage, which may be because IL-6 is involved in the promotion of osteoclast formation (33). Blocking IL-6 with tocilizumab (anti-IL-6 receptor (IL-6R) monoclonal antibody) can inhibit IgD^-^CD27^-^ memory B cells and significantly improve the clinical symptoms of RA patients (34, 35).

### IL-21

The cytokine IL-21 is produced by multiple helper T cell subsets, and has key functions in B cell activation, proliferation, differentiation, affinity maturation and antibody production. IL-21 drives the pro-inflammatory response by promoting B cell activation and expansion. Compared with healthy individuals, the concentration of IL-21 in the synovium and serum of RA patients is significantly increased (36). In addition, the proportion of IL-21R^+^ B cells in RA patients is also significantly higher than that in healthy people (37). In germinal center, IL-21 secreted by T follicular helper cells (Tfh) activates AID to regulate class switching of B cells and promote their differentiation into memory B cells and plasma cells (38). Therefore, blocking IL-21 will lead to the reduction of T cell-induced B cell proliferation and differentiation, and reduce the inflammatory response. In addition, IL-21R knockout mice are resistant to induction of CIA, indicating that IL-21 signaling in B cells is essential for the development of CIA (39). Treatment of CIA mice with IL-21R.Fc fusion protein can reduce their clinical signs, antibody levels and IL-6 levels, which also proves the important role of IL-21 in the development of CIA (40). New research shows that IL-21 can increase the binding of specificity protein 1 and IL21R promoter in B cells, leading to enhanced B cell response in RA patients (41). Since IL-21 promotes the function of B cells in RA patients through multiple mechanisms, targeting IL-21 as a treatment for RA may be valuable in the future.

### FcγRIIb

Fc gamma receptor IIβ (FcγRIIβ) is an inhibitory receptor that can inhibit BCR-mediated signal transduction and avoid excessive B cell activation (42). When BCR signal is activated by antigen, FcγRIIβ bound to BCR can further activate tyrosine kinase (Lyn) and immunoreceptor tyrosine based inhibitory motif (ITIM), and then recruit tyrosine phosphatase-1 (SHP-1) to inhibit signal downstream of BCR (43). Related studies have shown that FcγRIIβ helps prevent autoimmunity, and mutations in its genetic locus are related to RA (44). Hu C et al. found that YSTB (Yishen-tongbi decoction) can inhibit the excessive activation of B cells by activating the FcγRIIb/Lyn/SHP-1 pathway, thereby reducing the joint inflammation in CIA rats (45).

### Other Checkpoints

Other checkpoints, such as CD19, cytotoxic T lymphocyte-associated antigen-4 (CTLA4), programmed cell death 1 (PD1), also play important roles in the pathogenesis of B cells in RA (2, 46). As our understanding of their pathogenic mechanism gradually improves, these receptors can provide new targets for the future treatment of RA.

## B Cell Tolerance Checkpoints

**Figure 3:**
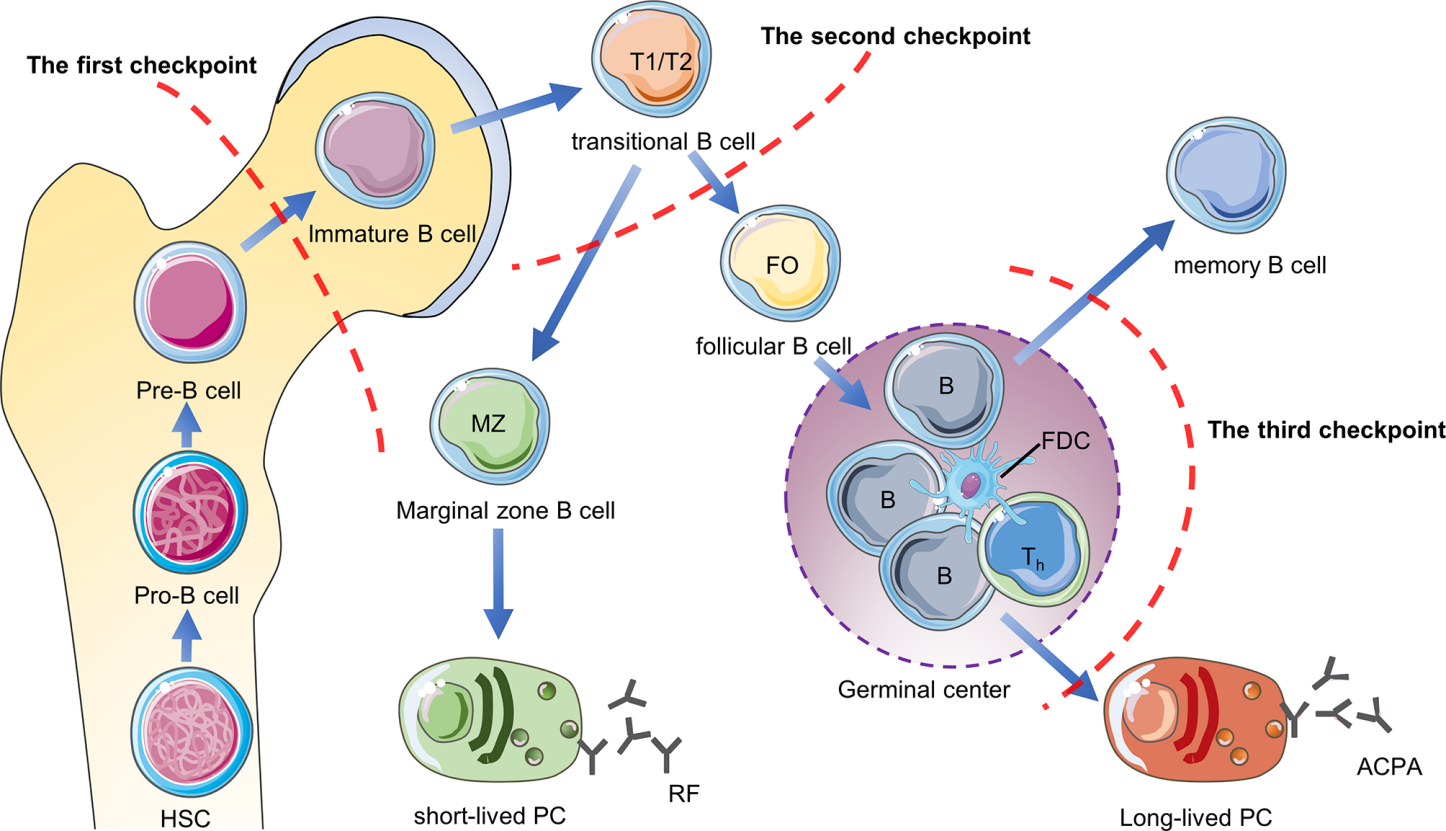
B cell tolerance checkpoints in RA. B cell tolerance checkpoints are “checkpoints” to reduce the number of autoreactive B cells in RA patients. After these checkpoints are impaired, a large number of autoreactive B cells will accumulate in RA patients and cause the production of autoantibodies such as RF and ACPA. HSC, hematopoietic stem cells; PC, plasma cells.

B cell tolerance checkpoints are mainly used to study self-reactive B cells, which refer to sites that regulate B cell tolerance and control the number of self-reactive B cells in the body during the development of B cells. These sites are distributed in the bone marrow, peripheral blood and germinal centers, but they have not been studied clearly. First of all, when the pre-B cells of the bone marrow develops to the immature B cells, they will be checked by the first tolerance checkpoint. Through clonal deletion, receptor-editing, anergy and other mechanisms, most of the autoreactive B cells in the bone marrow are eliminated (47). After immature B cells leave the bone marrow, they undergo a second tolerance checkpoint when they differentiate from new emigrant/transitional B cells into mature naive B cells in the blood and spleen Inspection. Through regulatory T cells to provide inhibitory signals and other mechanisms, the number of self-reactive B cells is further reduced (48). The mature naive B cells are activated after being stimulated by antigens, and the activated autoreactive B cells are corrected through co-stimulatory signals provided by T helper cells (T helper cells) and follicular dendritic cells (FDC) or somatic hypermutation (SHM) in the germinal center, thereby further reducing the production of autoreactive memory B cells and plasma cells (49, 50). However, some autoreactive B cells in RA patients have not been corrected after SHM. After they differentiate into plasma cells, they will secrete high-affinity ACPA and other autoantibodies, so they are more pathogenic (50, 51). The study found that compared with healthy individuals, the proportion of autoreactive new emigrant/transitional B cells and mature naive B cells in the peripheral blood of RA patients was significantly increased, indicating the central and peripheral B cell tolerance checkpoints of RA patients have been impaired (**Figure 3**).

Current research shows that ACPA-specific B cells and RF-specific B cells are the two main types of autoreactive B cells in RA (52). Mahendra et al. successfully isolated CCP^pos^ and CCP^neg^ B cells in the peripheral blood of RA patients and performed transcriptome sequencing (53). They found that compared with CCP^neg^ B cells, CCP^pos^ B cells highly expressed IL-15Rα, which may be a future therapeutic target for autoreactive B in RA patients (53). Germar et al. found that compared with CCP2^neg^ B cells, CCP2^pos^ B cells express high levels of CD40 and C5aR1 on the surface (54). C5aR1 may also be the surface markers of ACPA-specific B cells, but the sample size needs to be expanded for further confirmation. Tetramer technology is currently a good method for isolating autoreactive B cells in RA patients, which is beneficial to further research on B cell tolerance in RA patients in the future. The current research progress is limited to several mechanisms of how B cells escape the peripheral B cells tolerance checkpoint. The future research direction is to further clarify the mechanism of B cell tolerance checkpoint damage and to find biomarkers that distinguish between autoreactive B cells and non-autoreactive B cells, which will provide the basis for precise depletion of self-reactive B cells to treat RA.

## B Cells in the Pathogenesis of RA

**Figure 4:**
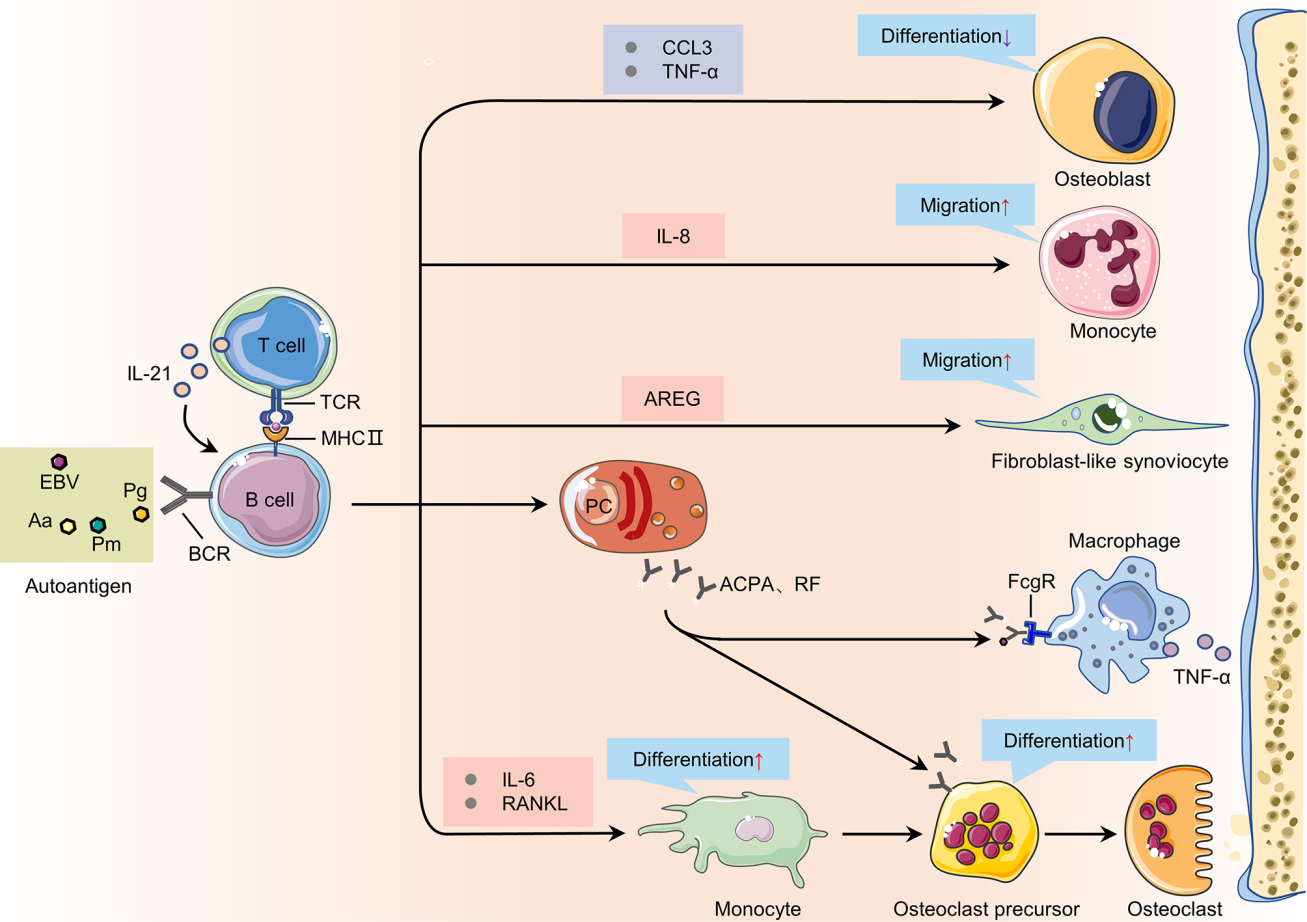
The immunopathogenic role of B cells in rheumatoid arthritis. During the onset of RA, B cells can promote the activation, proliferation, and differentiation of other cells such as T cells, monocytes, and osteoclasts in the synovium by providing cytokines, autoantibodies and other mediators. TCR, T cell receptor; MHC II, major histocompatibility complex class II; TNF-α, tumor necrosis factor-α; FcgR, Fcgamma receptors; AREG, amphiregulin; RANKL, receptor activator of nuclear factor kappa-B ligand; Pg, Porphyromonas gingivalis; Aa, Aggregatibacter actinomycetemcomitans; Pm, Proteus mirabilis; EBV, Epstein-Barr virus; PC, plasma cells.

The functions of B cells, including antigen presentation, cytokine secretion and autoantibody production, are all related to the pathogenesis of RA (**Figure 4**).

### Antigen Presentation

There are three main types of antigen-presenting cells in the human body: dendritic cells (DC), macrophages, and B cells. APC can rapidly endocytose, process and present antigens to CD4 ^+^ helper T cells to cause related immune responses. B cells are essential APCs in proteoglycan-induced arthritis (PGIA). In the early stage of CIA, autoreactive B cells may trigger or exacerbate the disease by activating autoreactive T cells. As the number of autoreactive B cells increases during inflammation, these B cells may play an increasingly important role in the activation of autoreactive T cells. In the PGIA model, PG-specific B cells are necessary to activate autoreactive T cells. In the absence of PG-specific B cells, the PG-specific T cells in the modeled mice cannot be activated, and eventually arthritis will not develop (55). Besides, citrullinated proteins are also major autoantigens that affects RA disease progression. Previous studies indicated that HLA-DRB1 alleles may bind citrullinated peptides and present them to T helper cells specific for citrullinated proteins (56). However, further researches show increased citrullination of self-antigens do not improve binding affinity for HLA-DR alleles and there is no evidence shows that citrullinated peptides preferentially bind to HLA-DR alleles (57, 58). Auger et al. found HLA-DR alleles could bind to PAD4 (an enzyme that binds and citrullinates multiple proteins) and use it as a carrier to internalize and process the PAD4-citrullinated protein complex, and present the PAD4 peptides to T helper cells, which could eventually lead to the production of IgG antibodies to multiple citrullinated proteins (59).

In RA, B cells, as APCs, mainly present their own antigens to CD4^+^T helper cells. CD4^+^ helper T cells are divided into follicular helper cells (Tfh) and peripheral helper cells (Tph). Compared with healthy individuals, Tfh cells and Tph cells in the synovium and peripheral blood of RA patients are significantly increased. Tfh cells and Tph cells can secrete CXCL13 and IL-21, and the latter is very important for the differentiation of B cells and the production of autoantibodies (60–62). After B cells present antigens to Tfh cells, Tfh cells can promote the affinity maturation of B cells. Several studies have observed that the proportion of CD4^+^ Tfh cells is positively correlated with serum ACPA titer in RA patients (63–65).

### Cytokine Secretion

In the synovium of RA patients, there is a complex network of cytokines, which are closely related to the occurrence of the disease. B cells in the peripheral blood of RA patients can secrete a variety of different cytokines to participate in bone destruction, including: TNF-α, IFN-γ, IL-6, IL-1β, IL-17 and IL-10 (66). Compared with ACPA negative RA patients, ACPA positive RA patients have significantly higher levels of IL-1β, CCL20, IL-17F and IL-10 in synovial fluid (67). After TLR9 and CD40 are activated, the amount of TNF-α produced by the B cells of RA patients is higher than that of healthy individuals (68). TNF-α can increase the expression of RANKL by B cells in the presence of IL-1β, thereby promoting the formation of osteoclasts (69). Sun W et al. found that B cells can also inhibit the differentiation of osteoblasts by producing TNF-α and CCL3 to inhibit bone formation in RA patients (70). IL-6 derived from B cells can promote its own proliferation and exert pleiotropic effects on T cells and other cells (71). RANKL is mainly secreted by memory B cells expressing Fc receptor like 4 (FcRL4) in the joints of RA patients, but these cells have low plasma cell differentiation potential (72, 73). In vitro experiments have shown that RANKL secreted by B cells can promote the differentiation of monocytes into osteoclasts, leading to bone damage in RA (11). IFN-γ secreted by B cells can maintain PGIA by promoting the production of autoreactive T cells and Tfh (74, 75). Kristyanto H et al. found that ACPA-positive B cells in the blood and synovial fluid of RA patients could secrete the chemokine interleukin 8 to attract neutrophils to the site of inflammation (76).

Regulatory B (Breg) cells are a type of B cells that exert immunosuppressive functions. In contrast to pro-inflammatory B cell responses, Breg cells are mainly responsible for the production of anti-inflammatory cytokines such as IL-10, TGFβ and IL-35. Breg cells can inhibit disease progression in RA, and the decrease in their number is related to the increase in disease activity of RA patients (77, 78). Human Breg cells are mainly enriched in transitional (CD19^+^CD24^hi^CD38^hi^) and memory (CD19^+^CD24^hi^CD27^+^) B cells (79). CD19^+^CD24^hi^CD38^hi^ B cells can inhibit the production of inflammatory factors such as IFN-γ and IL-21 by T cells in RA patients, while reducing the production of ACPA (80, 81). In addition, CD19^+^CD24^hi^CD27^+^ B cells derived from peripheral blood also play an important role in immune regulation and participate in inflammatory response (82). TGFβ is produced by some other Breg cells and also regulate T cell activity (83). In 2014, Shen P et al. described a group of Breg cells that suppress autoimmunity and secrete IL-35 (84, 85). There are two main regulatory B cell populations in mice: transitional B cells (CD19^+^CD21^hi^CD23^hi^CD1d^hi^) have been shown to prevent arthritis (86), and B10 B cells (CD19^+^CD5^+^CD1d^hi^) has been shown to maintain immune tolerance by inhibiting Th1/Th17 response and promoting Treg cell production in murine arthritis (87). Breg cells play an important role in alleviating the inflammatory response in RA patients, and how to restore or enhance the immunosuppressive function of Breg cells in RA patients still needs further research.

### Autoantibody Production

Autoantibodies are mainly secreted and produced by autoreactive B cells after they differentiate into plasma cells. The cross-reactivity of some post translational modification proteins and foreign antigens may drive the expansion of autoreactive B cells in RA (88). Current studies have confirmed that microorganisms from the intestines and lungs may induce the onset of RA. These microorganisms include Porphyromonas gingivalis (Pg) (89, 90), Aggregatibacter actinomycetemcomitans (Aa) (91), Proteus mirabilis (Pm) (92) and Epstein-Barr virus (EBV) (93). The autoantibodies of RA mainly include RF, ACPA, anti-modified citrullinated vimentin antibody, anti-carbamylated protein antibody, anti-PAD-4 antibody, anti-GPI antibody and so on (94). Many years before the onset of RA, autoantibodies such as RF and ACPA appeared in the patient’s serum (95). However, ACPA-specific B cells and RF-specific B cells have different developmental trajectories: ACPA-specific B cells undergo more rounds of germinal center reactions than RF-specific B cells (96). Compared with RF-specific B cells, ACPA-specific B cells have a higher proportion of somatic hypermutation and class switching (96). ACPA is present in approximately 70% of RA patients (97, 98), and compared with seronegative RA patients, patients positive for RF or ACPA have more severe disease progression (99, 100). ACPA is not limited to recognize citrullinated protein, but can also cross-react with acetylated and carbamylated proteins (88, 101, 102).

Autoantibodies such as RF and ACPA participate in the pathogenesis of RA through multiple mechanisms. In RA, immune complexes containing RF or ACPA activate the complement pathway, leading to the production of C5a and membrane attack complex, both of which can cause damage to the joints (103). The immune complex formed by RF and autoantigens can also induce osteoclast differentiation through Fcγ receptors (FcγR) to mediate bone destruction in RA patients (104). ACPAs are serum markers for the diagnosis of RA, and Approximately 90% of ACPA-IgG molecules carry N−glycans on the Fab-domain (105). In contrast to Fc glycans, these N−glycans on the Fab-domain are highly sialylated (106). N−glycans on the Fab-domain of ACPA-IgG can reduce the affinity to non-self antigens to provide survival advantages for autoreactive B cells (107). Sehnert et al. found that increasing the sialylation of IgG antibodies can reduced the number of CD138^+^/TACI^+^ plasma cells and CD19^+^ B cells in CIA mice to relieve their joint inflammation (108). In addition, the ACPA response in RA patients was characterized by extensive somatic hypermutation and limited avidity maturation (109, 110). Despite these advances, we are still uncertain how these characteristics are related to the process that eventually leads to arthritis. Mahendra et al. found that the combination of amphiregulin (AREG) produced by B cells and ACPA will further lead to osteoclast differentiation, which is the first comprehensive study on the transcriptome profile of ACPA-specific B cells and will serve as a resource to further investigate the role of autoreactive B cells in RA (53). Understanding the role of ACPA Fab-domain glycans in the development of ACPA-expressing B cells, together with the transcriptional profile of ACPA-specific B cells, will help us develop new therapies targeting autoreactive B cells in RA.

## B Cells in the Prevention of RA

In addition, we must be aware that not all B cells can promote the pathogenesis of RA, and that some antibodies produced by B cells have a preventive and protective effect on RA, such as naturally arising antibodies (NAbs) (111, 112), therapeutic anti-citrullinated protein antibodies (tACPAs) (113). In patients with autoimmune disease, higher levels of NAbs correlate with fewer cardiovascular events (114). A study in 2012 pointed out that compared with RA patients with high levels of IgM anti-phosphorylcholine NAbs, patients with low levels of NAbs had higher frequency of cardiovascular events within 5 years (112). In the experimental models, IgM NAbs can significantly reduce the clinical scores of their damaged joints and even prevent the development of inflammatory arthritis (114). The above studies fully demonstrate that antibodies produced by some B cells have a protective effect on RA. The capacity for NAbs influence pathogenesis of RA in people has not yet been directly examined and it remains to be further studied. tACPAs are also protective antibodies that specifically bind to citrulline at position 3 (Cit3) in histone 2A (citH2A) and 4 (citH4) (113). Compared with pathological ACPAs, tACPAs are extremely rare and extremely inferior in number (115). Therefore, ACPAs in RA patients mainly play pathogenic roles. A new research shows that tACPAs can diminish NET (neutrophil extracellular traps) release and potentially initiate NET uptake by macrophages *in vivo*, thereby reducing joints damage and disease progression in CIA mice (115). tACPAs opens up new avenues for the therapies for RA, but we still don’t know the difference between the B cells that produce pathological ACPA and tACPA, which may be very important for us to understand the role of B cells in RA development.

## The Prospect of Treating RA With B Cells as a Target

### Targets B cell Surface Receptors

**Figure 5:**
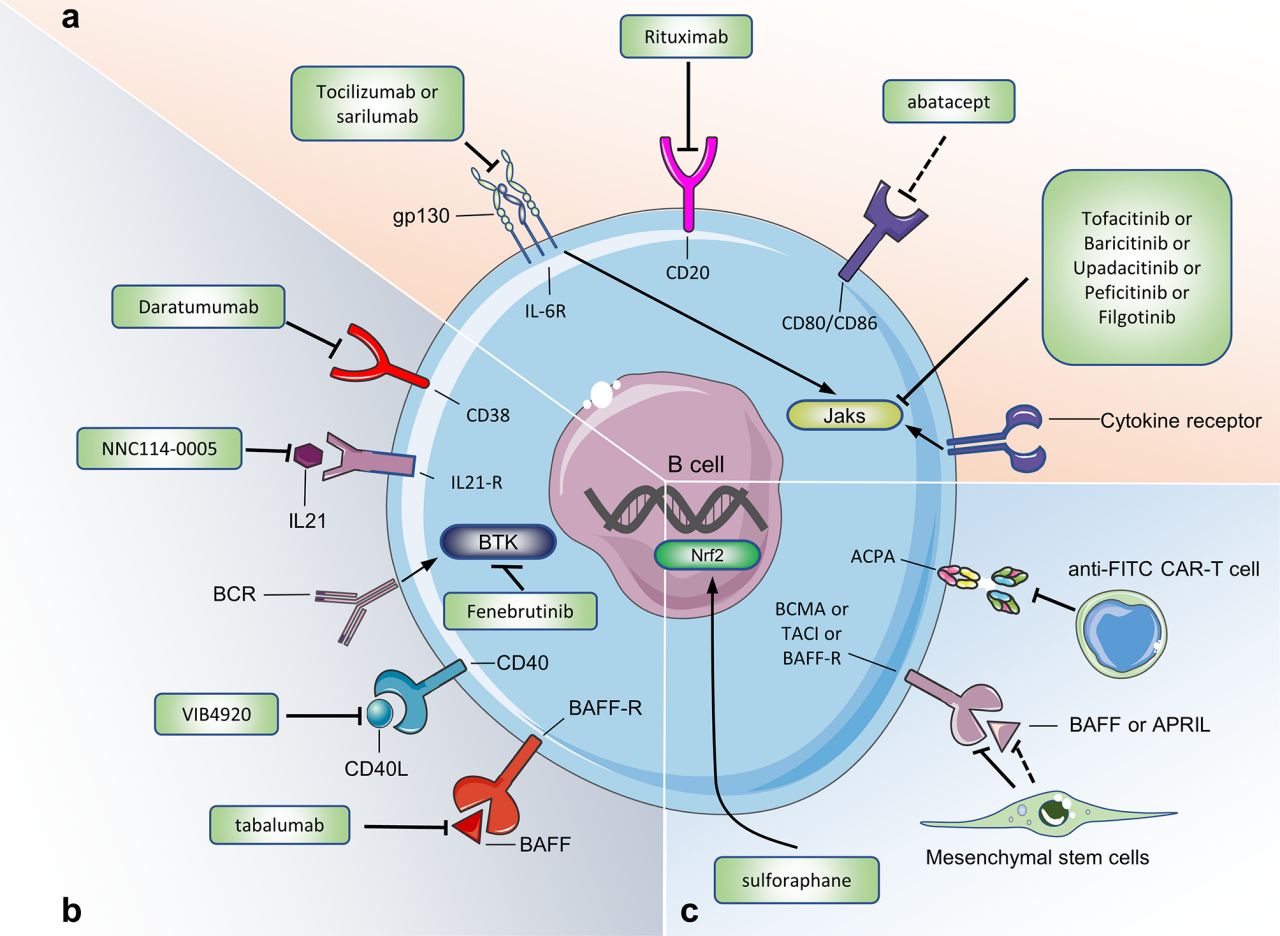
Targeting B cells for current and potential therapeutic approaches in RA. **(A)** Therapeutic approaches already approved in human. **(B)** Therapeutic approaches under evaluation in human. **(C)** Potential therapeutic approaches (only tested in mouse model of CIA or in experiments *in vitro*). **Figure 5** summarizes the drugs or cells that target B cells to treat RA. These drugs or cells are either approved in the clinical trial phase of RA, or we believe that they may be used for RA treatment. Jaks, Janus kinases; IL-21R, IL-21 receptor; IL-6R, IL-6 receptor; BAFFR, B cell activating factor receptor; BCMA, B cell maturation antigen; TACI, transmembrane activator and calcium modulator; BTK, Bruton’s tyrosine kinase; anti-FITC CAR-T cell, antifluorescein isothiocyanate chimeric antigen receptor T cells.

The use of Rituximab to deplete B cells is currently the most widely used treatment for treating RA with B cells as a target. CD20 is specifically expressed on the surface of 95% of human B cells. Rituximab targeting CD20 can deplete all B cells except pro-B cells and plasma cells (116, 117). RA patients treated with Rituximab showed positive clinical responses such as decreased synovial B cells, plasma cells, and IgG (118). However, pathogenic B cells and protective B cells treated with rituximab have been eliminated, which will cause a huge immunosuppressive effect in RA patients (**Figure 5**).

CD38 is mainly expressed on plasmablasts and plasma cells. In vitro experiments show that Daratumumab (an anti-CD38 monoclonal antibody) removes plasma cells and plasmablasts in PBMC of RA patients in a dose-dependent manner (119). Studies have reported that daratumumab has been successful in the treatment of 2 patients with refractory systemic lupus erythematosus, but the efficacy and safety of Daratumumab in the treatment of RA patients still need to be confirmed (120). Abatacept (CTLA-4Ig) has been successfully used to treat autoimmune diseases and has been approved for the treatment of RA. By binding to CD80 and CD86 on the surface of B cells, abatacept inhibits the co-stimulation and activation of T cells, leading to the down-regulation of inflammatory mediators. Studies have shown that Abatacept can inhibit the expression of CD80/CD86 on the surface of B cells in the peripheral blood of RA patients, while reducing the number of plasma cells and the level of serum IgG antibodies (46). In addition, the proportion of B cells in the synovium and ACPA-specific switched memory B-cells in the blood serum of RA patients decreased significantly after receiving Abatacept treatment (46, 121). ACPA-specific B cells are the main type of autoreactive B cells in RA patients. Co-culture experiments *in vitro* have shown that anti-FITC CAR-T cells can eliminate FITC-labeled ACPA-specific B cells (122). Whether this clearance effect exists in the body remains to be confirmed, but that study provides a new idea for the future use of CAR-T cell therapy to deplete autoreactive B cells to treat RA.

### Targeted B Cell Checkpoints

Bruton’s tyrosine kinase (BTK) is a cytoplasmic tyrosine kinase expressed in B cells, which plays a key role in BCR signal transduction and in the development and maturation of B cells (123). In the model of arthritis, BTK-deficient mice and BTK inhibitor-treated rodents showed reduced RA progression (124). A randomized, double-blind, phase II clinical trial of the BTK inhibitor Fenebrutinib in the treatment of RA patients (n = 578) showed that compared with the placebo group, the Fenebrutinib treatment group (1×150 mg/day, 2×200 mg/day) has a significant clinical effect (125). Other BTK inhibitors, such as Branebrutinib, are in phase 2 clinical trials for the treatment of RA patients. Nuclear factor E2-related factor 2 (Nrf2) is a transcription factor that plays an important role in cell resistance to oxidative damage. Moon et al. found that sulforaphane can inhibit B cell differentiation and antibody formation to reduce joint inflammation after activating Nrf2 in CIA mice (126).

Mesenchymal stem cells (MSCs) are a type of stem cells that have a wide range of sources, multiple differentiation potentials and immunomodulatory functions. They have been used in multiple clinical trials to treat RA (127). Experiments *in vitro* show that adipose tissue-derived MSCs co-cultured with peripheral blood B cells of RA patients can inhibit the proliferation of B cells and reduce the secretion of ACPA (128). Clinical trials have shown that MSCs can reduce joint inflammation by reducing the proportion of CD19^+^B cells and serum BAFF, APRIL and RF levels in RA patients (129). MSCs mainly act by secreting extracellular vesicles (including exosomes, Exos and microvesicles, MVs). Exos and MVs reduce the potential immune-related risks of MSCs and are a good substitute for MSCs. Cosenza et al. observed that MSC-derived Exos (with a diameter of less than 150 nm) can effectively reduce joint inflammation in CIA mice, including reducing the proportion of plasma cells and increasing the proportion of Breg cells in the peripheral blood, while reducing the level of IL-6, IL-1β, autoantibodies and increasing the level of IL-10 in the serum (130). Whether Exos derived from MSC has a similar effect on B cells in RA patients remains to be confirmed. In short, MSCs and Exos derived from MSCs have broad application prospects in reducing joint inflammation and repairing the immune function of B cells in RA.

### Targeting B Cell Related Cytokines

The use of TNF inhibitors can significantly reduce the level of IgD^-^CD27^-^ B cells, while increasing the level of Breg cells in RA patients (35, 131). Belimumab and Tabalumab are two anti-BAFF biological agents. Clinical studies have shown that, compared with the placebo group, Belimumab shows better efficacy in the treatment of RA patients with RF^+^, ACPA^+^, DAS28>5.1 (132). In addition, compared with the placebo group, the number of RA patients treated with 120 mg of Tabalumab was significantly higher when the ACR20 and ACR50 response rates were reached (133). Long-term treatment with Tabalumab can cause a decrease in total B cells, mature naive B cells, and switched memory B cells in RA patients (134). However, the phase III clinical trial of Tabalumab in the treatment of RA was forced to stop because the interim results did not meet the expected efficacy (135). RA patients who received Atacicept (a biological agent that blocks the combination of BAFF/APRIL and TACI) showed a significant reduction in serum anti-RF levels, but in phase II clinical trials, Atacicept did not show significant clinical improvement compared with the placebo group (136).

Targeting IL-6 has shown efficacy in the treatment of various autoimmune diseases. At present, the anti-IL-6R monoclonal antibodies (tocilizumab and sarilumab) have been approved for the treatment of RA and have shown good efficacy (137). Tocilizumab may reduce the serum ACPA titer of RA patients by increasing the ratio of post-switch memory B cells (IgD-CD27+)/mature naive B cells (138, 138). In a randomized, double-blind clinical trial for RA, NNC114-0005 (anti-IL-21 Monoclonal antibodies) can reduce the disease activity of RA patients and neutralize IL-21 in their bodies (139). Based on the results of that clinical trial, we can further explore the effects of IL-21 as a target in the treatment of RA.

Janus Kinase (JAK) mediates signal transduction through IL-6R and many other transmembrane receptors (cytokine receptors, G protein-coupled receptors, receptor tyrosine kinases). JAK inhibitors can block the effects of pro-inflammatory cytokines on B cells (140), and five JAK inhibitors (tofacitinib, upadacitinib, baricitinib, peficitinib, filgotinib) have been approved for the treatment of RA. Tofacitinib and upadacitinib inhibit B cell proliferation and activation by blocking signal transduction mediated by JAK1 and JAK3 (141–143). The use of tofacitinib significantly reduce the levels of RF in the peripheral blood of RA patient (144). Baricitinib can inhibit the differentiation of B cells into plasmablasts and inhibit the production of IL-6 (145). Similar to tofacitinib, baricitinib reduces the expression of BAFF in RA synovial fibroblasts, thereby locally inhibiting B cell activation in joints (146). Filgotinib directly inhibits human B cell differentiation and IgG production. After RA patients received filgotinib treatment, the representative of B cell chemotaxis [chemokine (CXC motif) ligand 13, CXCL13], survival and activation (BAFF), differentiation (IL-2, IL-5, IL-7, IL-21) are significantly reduced (147). In short, JAK inhibitors can treat RA by inhibiting B cell activation, proliferation and differentiation, but further studies are still needed to clarify the exact mechanism of action of JAK inhibitors on B cells and other immune cells.

## Conclusion

More and more data show that B cells promote the pathogenesis of RA through a variety of mechanisms. As our understanding of B cells in the pathogenesis of RA gradually improves, we must realize that not all B cells in RA patients are pathogenic. On the one hand, we need to continue to study the mechanism by which autoreactive B cells escape the B cell tolerance checkpoints, so as to further understand how pathogenic B cells are produced in RA patients. On the other hand, how to distinguish between pathogenic B cells and protective B cells will become an important direction for precise treatment of RA. In the future, it is very likely that therapies against B cells that produce autoantibodies will be developed to precisely target pathogenic B cells.

## Author Contributions

This article is mainly written by FW. JG and JK wrote part of the manuscript and proofread the manuscript. XW, QN, and JL helped us collect literature information and draw pictures. LZ reviewed the manuscript and proposed final revisions. All authors contributed to the article and approved the submitted version.

## Funding

This work was supported by the National Natural Science Foundation of China [grant number 81771768] and by the applied basic research project of Shanxi Science and Technology Department [grant number 201901D111416].

## Conflict of Interest

The authors declare that the research was conducted in the absence of any commercial or financial relationships that could be construed as a potential conflict of interest.

## Publisher’s Note

All claims expressed in this article are solely those of the authors and do not necessarily represent those of their affiliated organizations, or those of the publisher, the editors and the reviewers. Any product that may be evaluated in this article, or claim that may be made by its manufacturer, is not guaranteed or endorsed by the publisher.
